# Crystal structure of triethyl 2-(5-nitro-2*H*-indazol-2-yl)propane-1,2,3-tri­carboxyl­ate

**DOI:** 10.1107/S2056989015017235

**Published:** 2015-09-26

**Authors:** Mohammed Boulhaoua, Mohammed Benchidmi, El Mokhtar Essassi, Mohamed Saadi, Lahcen El Ammari

**Affiliations:** a Laboratoire de Chimie Organique Hétérocyclique URAC 21, Pôle de Compétence Pharmacochimie, Av. Ibn Battouta, BP 1014, Faculté des Sciences, Université Mohammed V, Rabat, Morocco; b Laboratoire de Chimie du Solide Appliquée, Faculté des Sciences, Université Mohammed V, Avenue Ibn Battouta, BP 1014, Rabat, Morocco

**Keywords:** crystal structure, indazole, ester, pharmacological properties, biochemical properties

## Abstract

In the title compound, C_19_H_23_N_3_O_8_, the 5-nitro-2*H*-indazol-2-yl unit is almost planar, with the maximum deviation from the mean plane being 0.024 (2) Å. The fused-ring system is nearly perpendicular to the three carboxyl­ate groups, with dihedral angles of 90.0 (3), 83.8 (1) and 80.4 (1)°. The ethyl groups attached to both ends of the propane chain are each disordered over two sets of sites, with site-occupancy ratios of 0.425 (17):0.575 (17) and 0.302 (15):0.698 (15). In the crystal, mol­ecules are linked by pairs of C—H⋯N hydrogen bonds, forming inversion dimers. The dimers are further linked by C—H⋯O hydrogen bonds, forming a three-dimensional network.

## Related literature   

For the pharmacological and biochemical properties of indazoles and their derivatives, see: Abbassi *et al.* (2014 ▸); Li *et al.* (2003 ▸); Lee *et al.* (2001 ▸). For compounds with similar structures, see: El Brahmi *et al.* (2012 ▸); Chicha *et al.* (2013 ▸).
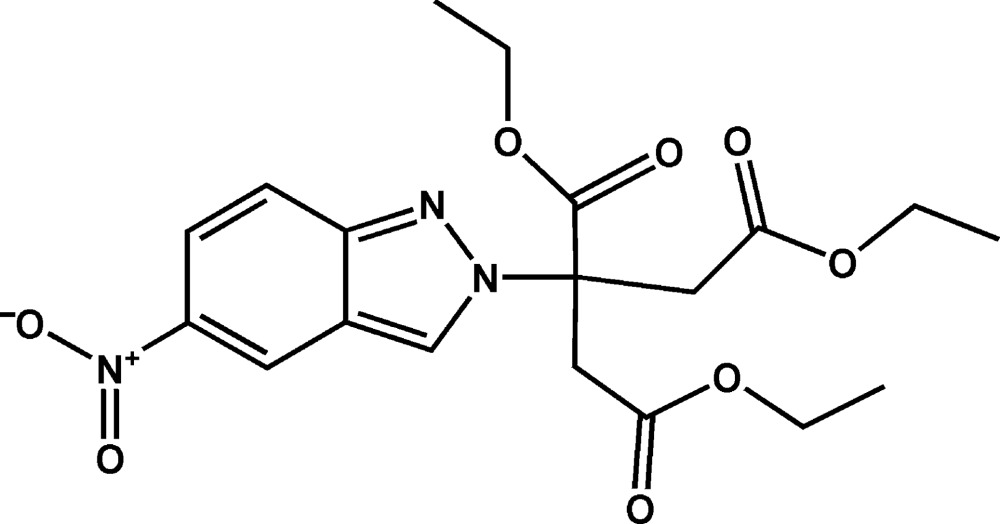


## Experimental   

### Crystal data  

C_19_H_23_N_3_O_8_
*M* *_r_* = 421.40
Monoclinic, 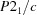

*a* = 13.4555 (4) Å
*b* = 18.6185 (6) Å
*c* = 8.5258 (3) Å
β = 104.603 (1)°
*V* = 2066.90 (12) Å^3^
*Z* = 4
Mo *K*α radiation
μ = 0.11 mm^−1^
*T* = 296 K
0.42 × 0.31 × 0.26 mm

### Data collection  

Bruker X8 APEX diffractometer
Absorption correction: multi-scan (*SADABS*; Bruker, 2009 ▸) *T*_min_ = 0.673, *T*_max_ = 0.746
36533 measured reflections
4233 independent reflections
3523 reflections with *I* > 2σ(*I*)
*R*_int_ = 0.031

### Refinement  

*R*[*F* ^2^ > 2σ(*F* ^2^)] = 0.045
*wR*(*F* ^2^) = 0.129
*S* = 1.03
4233 reflections
309 parameters
4 restraints
H-atom parameters constrained
Δρ_max_ = 0.30 e Å^−3^
Δρ_min_ = −0.25 e Å^−3^

### 

Data collection: *APEX2* (Bruker, 2009 ▸); cell refinement: *SAINT* (Bruker, 2009 ▸); data reduction: *SAINT*; program(s) used to solve structure: *SHELXS2013* (Sheldrick, 2008 ▸); program(s) used to refine structure: *SHELXL2013* (Sheldrick, 2008 ▸); molecular graphics: *ORTEP-3 for Windows* (Farrugia, 2012 ▸); software used to prepare material for publication: *PLATON* (Spek, 2009 ▸) and *publCIF* (Westrip, 2010 ▸).

## Supplementary Material

Crystal structure: contains datablock(s) I, global. DOI: 10.1107/S2056989015017235/is5422sup1.cif

Structure factors: contains datablock(s) I. DOI: 10.1107/S2056989015017235/is5422Isup2.hkl

Supporting information file. DOI: 10.1107/S2056989015017235/is5422Isup3.cml

**Fig. 1.**
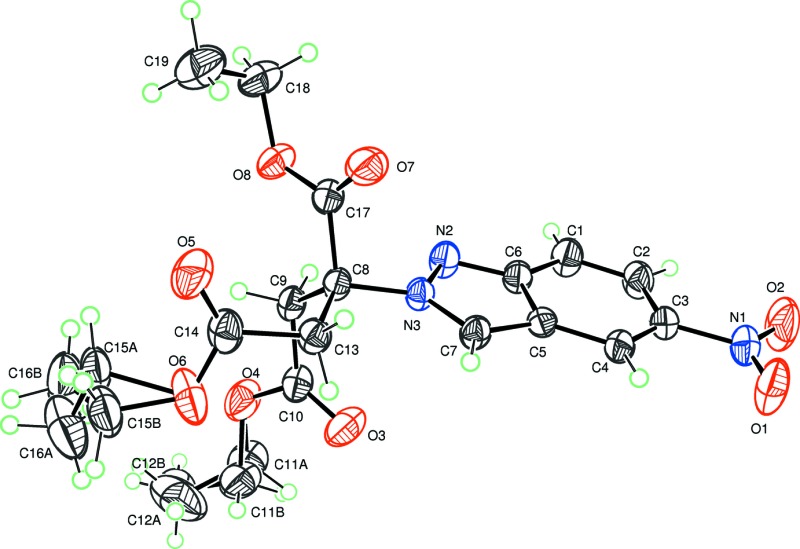
Molecular structure of the title compound with the atom-labelling scheme. Displacement ellipsoids are drawn at the 50% probability level. H atoms are represented as small circles.

. DOI: 10.1107/S2056989015017235/is5422fig1.tif
Mol­ecular structure of the title compound with the atom-labelling scheme. Displacement ellipsoids are drawn at the 50% probability level. H atoms are represented as small circles.

**Fig. 2.**
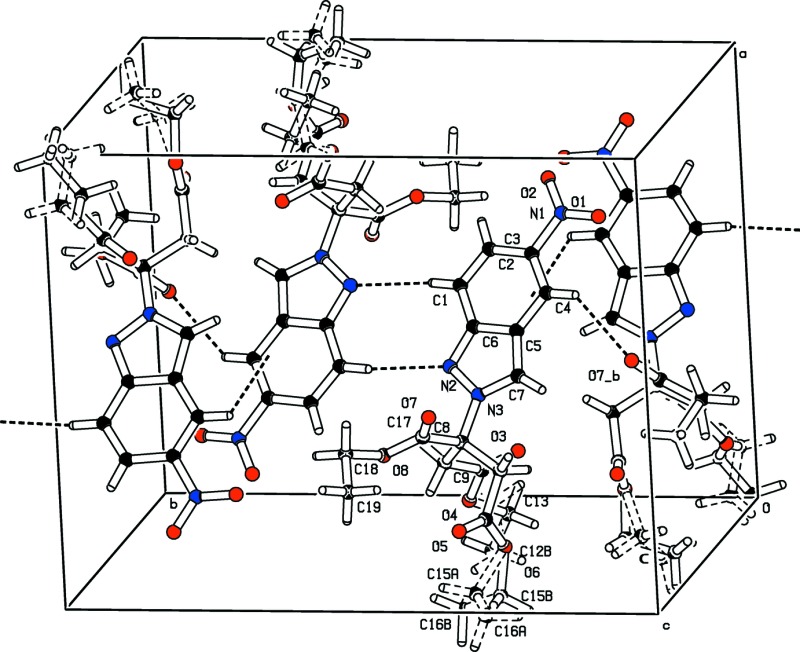
A packing view of the title compound showing molecules linked together by hydrogen bonds as dashed lines.

. DOI: 10.1107/S2056989015017235/is5422fig2.tif
A packing view of the title compound showing mol­ecules linked together by hydrogen bonds as dashed lines.

CCDC reference: 1424508

Additional supporting information:  crystallographic information; 3D view; checkCIF report

## Figures and Tables

**Table 1:** Hydrogen-bond geometry (, )

*D*H*A*	*D*H	H*A*	*D* *A*	*D*H*A*
C1H1N2^i^	0.93	2.53	3.443(2)	168
C4H4O7^ii^	0.93	2.53	3.205(2)	129

Symmetry codes: (i) 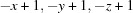; (ii) 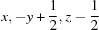.

## supplementary crystallographic information

### S1. Comment

Recently, a number of pharmacological tests revealed that the indazole derivatives present various biological activities and other chemical industries. With respect to biological applications, these derivatives are found potent anti-tumour (Abbassi *et al.*, 2014), anti-microbial (Li *et al.*, 2003) and anti-platelet (Lee *et al.*, 2001). As a continuation of our research work devoted to the development of *N*-substituted indazole and trying potential pharmacological activities (El Brahmi *et al.*, 2012; Chicha *et al.*, 2013), we have studied the reaction of ethyl bromoacetate towards 5-nitro-1*H*-indazole under phase-transfer catalysis conditions using tetra-*n*-butylammonium iodide (TBAI) as catalyst and potassium carbonate as base. The title compound was isolated and its structure was established.

The molecule of the title compound is build up from two fused five- and six-membered rings linked to a nitro group and to propane-1,2,3-tricarboxylate, which are connected to three ethyl moieties as shown in Fig. 1. The two ethyl groups attached to both ends of the propane chain are each split with occupancy ratios of 0.425 (17) (Cl1A–C12A) : 0.575 (17) (Cl1B–C12B) and 0.302 (15) (C15A–C16A) : 0.698 (15) (C15B–C16B). The fused ring system is virtually planar with the largest deviation from the mean plane being 0.013 (2) Å at N2 and makes dihedral angles of 83.8 (1), 80.5 (1) and 90.0 (3)° with the three carboxylate groups (C10/O3/O4), (C14/O/5O6) and (C17/O7/O8), respectively. In the crystal, the molecules are linked together by non-classic hydrogen bonds, forming a three dimensional network (Fig. 2 and Table 1).

### S2. Experimental

To a solution of 5-nitro-1*H*-indazole (0.5 g, 3 mmol) in THF (30 ml) was added ethyl bromoacetate (2 g, 12 mmol), potassium carbonate (1.24 g, 9 mmol) and a catalytic quantity of tetra-*n*-butylammonium iodide. The mixture was stirred at room temperature for 48 h. The solution was filtered and the solvent removed under reduced pressure. The residue was recrystallized from ethanol to afford the title compound as colourless crystals (yield: 66%; m.p. = 381 K).

### S3. Refinement

The H atoms were located in a difference map and treated as riding with C—H = 0.93 Å (aromatic), 0.97 Å (methylene) and 0.96 Å (methyl), and with *U*_iso_(H) = 1.2*U*_eq_ (aromatic and methylene C) and 1.5*U*_eq_(methyl C). The two ethyl groups attached to both ends of the propane chain are disordered with occupancy ratios of 0.425 (17):0.575 (17) and 0.302 (15):0.698 (15). The C11A—C12A, C11B—C12B, C15A—C16A and C15A—C16B distances were restrained to 1.483 (1) Å.

### Crystal data

**Table:**

C_19_H_23_N_3_O_8_	*F*(000) = 888
*M**_r_* = 421.40	*D*_x_ = 1.354 Mg m^−^^3^
Monoclinic, *P*2_1_/*c*	Mo *K*α radiation, λ = 0.71073 Å
*a* = 13.4555 (4) Å	Cell parameters from 4233 reflections
*b* = 18.6185 (6) Å	θ = 2.7–26.4°
*c* = 8.5258 (3) Å	µ = 0.11 mm^−^^1^
β = 104.603 (1)°	*T* = 296 K
*V* = 2066.90 (12) Å^3^	Block, colourless
*Z* = 4	0.42 × 0.31 × 0.26 mm

### Data collection

**Table:**

Bruker X8 APEX diffractometer	4233 independent reflections
Radiation source: fine-focus sealed tube	3523 reflections with *I* > 2σ(*I*)
Graphite monochromator	*R*_int_ = 0.031
φ and ω scans	θ_max_ = 26.4°, θ_min_ = 2.7°
Absorption correction: multi-scan (*SADABS*; Bruker, 2009)	*h* = −16→16
*T*_min_ = 0.673, *T*_max_ = 0.746	*k* = −23→23
36533 measured reflections	*l* = −10→10

### Refinement

**Table:**

Refinement on *F*^2^	4 restraints
Least-squares matrix: full	Hydrogen site location: inferred from neighbouring sites
*R*[*F*^2^ > 2σ(*F*^2^)] = 0.045	H-atom parameters constrained
*wR*(*F*^2^) = 0.129	*w* = 1/[σ^2^(*F*_o_^2^) + (0.0586*P*)^2^ + 0.8225*P*] where *P* = (*F*_o_^2^ + 2*F*_c_^2^)/3
*S* = 1.03	(Δ/σ)_max_ = 0.003
4233 reflections	Δρ_max_ = 0.30 e Å^−^^3^
309 parameters	Δρ_min_ = −0.25 e Å^−^^3^

### Special details

**Table:**

Geometry. All e.s.d.'s (except the e.s.d. in the dihedral angle between two l.s. planes) are estimated using the full covariance matrix. The cell e.s.d.'s are taken into account individually in the estimation of e.s.d.'s in distances, angles and torsion angles; correlations between e.s.d.'s in cell parameters are only used when they are defined by crystal symmetry. An approximate (isotropic) treatment of cell e.s.d.'s is used for estimating e.s.d.'s involving l.s. planes.

### Fractional atomic coordinates and isotropic or equivalent isotropic displacement parameters (Å^2^)

**Table:**

	*x*	*y*	*z*	*U*_iso_*/*U*_eq_	Occ. (<1)
C1	0.57273 (14)	0.40719 (9)	0.4318 (2)	0.0514 (4)	
H1	0.5740	0.4566	0.4170	0.062*	
C2	0.63996 (13)	0.36345 (10)	0.3833 (2)	0.0510 (4)	
H2	0.6882	0.3830	0.3344	0.061*	
C3	0.63713 (11)	0.28818 (9)	0.4066 (2)	0.0423 (4)	
C4	0.56810 (11)	0.25484 (8)	0.4740 (2)	0.0392 (3)	
H4	0.5675	0.2052	0.4859	0.047*	
C5	0.49768 (11)	0.29933 (8)	0.52479 (18)	0.0340 (3)	
C6	0.50052 (11)	0.37504 (8)	0.5058 (2)	0.0385 (3)	
C7	0.41751 (11)	0.28790 (8)	0.59778 (19)	0.0360 (3)	
H7	0.3948	0.2439	0.6272	0.043*	
C8	0.29510 (11)	0.37241 (8)	0.69122 (19)	0.0352 (3)	
C9	0.22024 (12)	0.42301 (8)	0.5780 (2)	0.0395 (3)	
H9A	0.1699	0.4399	0.6332	0.047*	
H9B	0.2579	0.4645	0.5551	0.047*	
C10	0.16487 (13)	0.38884 (9)	0.4201 (2)	0.0451 (4)	
C11A	0.0301 (7)	0.4160 (8)	0.1824 (13)	0.074 (3)	0.425 (17)
H11A	0.0590	0.3764	0.1346	0.088*	0.425 (17)
H11B	0.0330	0.4596	0.1212	0.088*	0.425 (17)
C12A	−0.0758 (10)	0.4003 (13)	0.194 (2)	0.130 (6)	0.425 (17)
H12A	−0.1191	0.3917	0.0876	0.195*	0.425 (17)
H12B	−0.1018	0.4405	0.2419	0.195*	0.425 (17)
H12C	−0.0752	0.3585	0.2601	0.195*	0.425 (17)
C11B	0.0141 (6)	0.3852 (5)	0.2097 (12)	0.075 (2)	0.575 (17)
H11C	0.0492	0.3815	0.1236	0.090*	0.575 (17)
H11D	−0.0005	0.3371	0.2415	0.090*	0.575 (17)
C12B	−0.0826 (6)	0.4264 (6)	0.1532 (14)	0.089 (3)	0.575 (17)
H12D	−0.1247	0.4041	0.0579	0.134*	0.575 (17)
H12E	−0.0669	0.4747	0.1281	0.134*	0.575 (17)
H12F	−0.1187	0.4272	0.2369	0.134*	0.575 (17)
C13	0.24285 (12)	0.30296 (8)	0.7280 (2)	0.0393 (3)	
H13A	0.2198	0.2751	0.6293	0.047*	
H13B	0.2920	0.2742	0.8055	0.047*	
C14	0.15273 (14)	0.32020 (10)	0.7957 (2)	0.0503 (4)	
C15A	−0.0178 (6)	0.3478 (8)	0.7695 (11)	0.057 (3)	0.302 (15)
H15A	−0.0369	0.3170	0.8488	0.069*	0.302 (15)
H15B	0.0080	0.3931	0.8198	0.069*	0.302 (15)
C16A	−0.1043 (10)	0.3585 (16)	0.6243 (16)	0.107 (7)	0.302 (15)
H16A	−0.1639	0.3741	0.6574	0.161*	0.302 (15)
H16B	−0.1191	0.3140	0.5661	0.161*	0.302 (15)
H16C	−0.0858	0.3942	0.5555	0.161*	0.302 (15)
C15B	−0.0333 (4)	0.3135 (4)	0.7256 (9)	0.0766 (16)	0.698 (15)
H15C	−0.0248	0.3055	0.8406	0.092*	0.698 (15)
H15D	−0.0797	0.2773	0.6663	0.092*	0.698 (15)
C16B	−0.0756 (7)	0.3862 (2)	0.6799 (14)	0.106 (3)	0.698 (15)
H16D	−0.1443	0.3888	0.6929	0.158*	0.698 (15)
H16E	−0.0763	0.3957	0.5690	0.158*	0.698 (15)
H16F	−0.0335	0.4213	0.7485	0.158*	0.698 (15)
C17	0.34844 (12)	0.40826 (9)	0.8553 (2)	0.0423 (4)	
C18	0.35337 (18)	0.50899 (12)	1.0282 (3)	0.0682 (6)	
H18A	0.4263	0.4989	1.0649	0.082*	
H18B	0.3451	0.5605	1.0131	0.082*	
C19	0.3021 (2)	0.48556 (16)	1.1524 (3)	0.0839 (7)	
H19A	0.3314	0.5103	1.2521	0.126*	
H19B	0.3112	0.4347	1.1690	0.126*	
H19C	0.2301	0.4963	1.1172	0.126*	
N1	0.71300 (11)	0.24372 (9)	0.3546 (2)	0.0561 (4)	
N2	0.42831 (10)	0.40789 (7)	0.56357 (18)	0.0432 (3)	
N3	0.37965 (9)	0.35279 (6)	0.61715 (15)	0.0346 (3)	
O1	0.71038 (13)	0.17910 (8)	0.3693 (2)	0.0810 (5)	
O2	0.77500 (13)	0.27406 (10)	0.2951 (3)	0.0952 (6)	
O3	0.19189 (12)	0.33658 (8)	0.36163 (16)	0.0686 (4)	
O4	0.08034 (11)	0.42484 (9)	0.35264 (18)	0.0758 (5)	
O5	0.16075 (13)	0.34111 (10)	0.9308 (2)	0.0840 (5)	
O6	0.06455 (10)	0.30905 (10)	0.6864 (2)	0.0762 (5)	
O7	0.41569 (11)	0.37912 (8)	0.95228 (17)	0.0683 (4)	
O8	0.31115 (10)	0.47256 (6)	0.87260 (15)	0.0528 (3)	

### Atomic displacement parameters (Å^2^)

**Table:**

	*U*^11^	*U*^22^	*U*^33^	*U*^12^	*U*^13^	*U*^23^
C1	0.0514 (10)	0.0374 (8)	0.0739 (12)	−0.0036 (7)	0.0316 (9)	0.0027 (8)
C2	0.0439 (9)	0.0520 (10)	0.0651 (12)	−0.0052 (7)	0.0285 (8)	−0.0004 (8)
C3	0.0330 (7)	0.0481 (9)	0.0468 (9)	0.0030 (6)	0.0119 (7)	−0.0071 (7)
C4	0.0356 (7)	0.0352 (7)	0.0467 (9)	0.0034 (6)	0.0105 (7)	−0.0024 (6)
C5	0.0322 (7)	0.0320 (7)	0.0378 (8)	−0.0002 (5)	0.0083 (6)	−0.0012 (6)
C6	0.0372 (7)	0.0327 (7)	0.0484 (9)	−0.0008 (6)	0.0160 (7)	−0.0011 (6)
C7	0.0378 (7)	0.0283 (7)	0.0439 (8)	0.0015 (6)	0.0138 (6)	0.0009 (6)
C8	0.0351 (7)	0.0319 (7)	0.0418 (8)	0.0014 (6)	0.0159 (6)	−0.0019 (6)
C9	0.0394 (8)	0.0355 (7)	0.0467 (9)	0.0060 (6)	0.0169 (7)	0.0002 (6)
C10	0.0455 (9)	0.0487 (9)	0.0429 (9)	0.0099 (7)	0.0148 (7)	0.0025 (7)
C11A	0.077 (5)	0.086 (7)	0.051 (4)	0.022 (4)	0.004 (3)	0.000 (4)
C12A	0.091 (9)	0.213 (17)	0.077 (8)	−0.061 (9)	0.004 (6)	−0.018 (9)
C11B	0.068 (4)	0.084 (5)	0.058 (4)	0.025 (3)	−0.013 (3)	−0.017 (3)
C12B	0.065 (4)	0.116 (5)	0.077 (5)	0.025 (3)	−0.003 (3)	−0.002 (3)
C13	0.0410 (8)	0.0362 (8)	0.0442 (9)	−0.0024 (6)	0.0172 (7)	0.0010 (6)
C14	0.0526 (10)	0.0478 (9)	0.0591 (11)	−0.0020 (8)	0.0302 (9)	0.0079 (8)
C15A	0.042 (4)	0.066 (7)	0.073 (5)	0.001 (4)	0.031 (4)	0.001 (4)
C16A	0.050 (6)	0.177 (19)	0.095 (9)	0.008 (8)	0.020 (5)	0.029 (10)
C15B	0.048 (2)	0.082 (3)	0.111 (4)	−0.005 (2)	0.039 (2)	−0.005 (3)
C16B	0.078 (5)	0.103 (4)	0.152 (8)	0.031 (3)	0.058 (5)	0.012 (4)
C17	0.0384 (8)	0.0414 (8)	0.0496 (9)	−0.0001 (6)	0.0156 (7)	−0.0072 (7)
C18	0.0766 (14)	0.0605 (12)	0.0694 (14)	−0.0083 (10)	0.0222 (11)	−0.0304 (10)
C19	0.0993 (18)	0.0977 (18)	0.0555 (13)	0.0002 (15)	0.0214 (13)	−0.0182 (12)
N1	0.0420 (8)	0.0628 (10)	0.0689 (11)	0.0059 (7)	0.0240 (7)	−0.0085 (8)
N2	0.0454 (7)	0.0288 (6)	0.0626 (9)	−0.0017 (5)	0.0269 (7)	0.0016 (6)
N3	0.0353 (6)	0.0278 (6)	0.0440 (7)	0.0005 (5)	0.0163 (5)	0.0008 (5)
O1	0.0826 (11)	0.0582 (9)	0.1184 (14)	0.0257 (8)	0.0552 (10)	0.0049 (9)
O2	0.0723 (10)	0.0863 (12)	0.1535 (18)	−0.0031 (9)	0.0777 (12)	−0.0181 (11)
O3	0.0801 (10)	0.0674 (9)	0.0513 (8)	0.0270 (7)	0.0039 (7)	−0.0158 (7)
O4	0.0639 (9)	0.0976 (11)	0.0563 (8)	0.0383 (8)	−0.0025 (7)	−0.0173 (8)
O5	0.0877 (11)	0.1046 (13)	0.0768 (11)	−0.0112 (9)	0.0526 (9)	−0.0237 (9)
O6	0.0411 (7)	0.1151 (13)	0.0780 (10)	0.0068 (7)	0.0252 (7)	0.0200 (9)
O7	0.0600 (8)	0.0730 (9)	0.0603 (8)	0.0205 (7)	−0.0067 (7)	−0.0192 (7)
O8	0.0645 (8)	0.0419 (6)	0.0531 (7)	0.0044 (5)	0.0169 (6)	−0.0119 (5)

### Geometric parameters (Å, º)

**Table:**

C1—C2	1.357 (2)	C12B—H12D	0.9600
C1—C6	1.418 (2)	C12B—H12E	0.9600
C1—H1	0.9300	C12B—H12F	0.9600
C2—C3	1.417 (2)	C13—C14	1.503 (2)
C2—H2	0.9300	C13—H13A	0.9700
C3—C4	1.359 (2)	C13—H13B	0.9700
C3—N1	1.467 (2)	C14—O5	1.195 (2)
C4—C5	1.407 (2)	C14—O6	1.327 (2)
C4—H4	0.9300	C15A—C16A	1.4828 (10)
C5—C7	1.391 (2)	C15A—O6	1.627 (10)
C5—C6	1.420 (2)	C15A—H15A	0.9700
C6—N2	1.3424 (19)	C15A—H15B	0.9700
C7—N3	1.3375 (18)	C16A—H16A	0.9600
C7—H7	0.9300	C16A—H16B	0.9600
C8—N3	1.4794 (18)	C16A—H16C	0.9600
C8—C9	1.531 (2)	C15B—O6	1.440 (4)
C8—C13	1.541 (2)	C15B—C16B	1.4825 (10)
C8—C17	1.553 (2)	C15B—H15C	0.9700
C9—C10	1.506 (2)	C15B—H15D	0.9700
C9—H9A	0.9700	C16B—H16D	0.9600
C9—H9B	0.9700	C16B—H16E	0.9600
C10—O3	1.191 (2)	C16B—H16F	0.9600
C10—O4	1.320 (2)	C17—O7	1.191 (2)
C11A—O4	1.448 (11)	C17—O8	1.3205 (19)
C11A—C12A	1.4826 (10)	C18—C19	1.468 (3)
C11A—H11A	0.9700	C18—O8	1.471 (2)
C11A—H11B	0.9700	C18—H18A	0.9700
C12A—H12A	0.9600	C18—H18B	0.9700
C12A—H12B	0.9600	C19—H19A	0.9600
C12A—H12C	0.9600	C19—H19B	0.9600
C11B—C12B	1.4824 (10)	C19—H19C	0.9600
C11B—O4	1.508 (8)	N1—O1	1.211 (2)
C11B—H11C	0.9700	N1—O2	1.219 (2)
C11B—H11D	0.9700	N2—N3	1.3567 (17)
			
C2—C1—C6	117.83 (15)	C14—C13—C8	110.65 (13)
C2—C1—H1	121.1	C14—C13—H13A	109.5
C6—C1—H1	121.1	C8—C13—H13A	109.5
C1—C2—C3	120.47 (15)	C14—C13—H13B	109.5
C1—C2—H2	119.8	C8—C13—H13B	109.5
C3—C2—H2	119.8	H13A—C13—H13B	108.1
C4—C3—C2	123.80 (14)	O5—C14—O6	125.14 (18)
C4—C3—N1	118.15 (15)	O5—C14—C13	123.66 (18)
C2—C3—N1	118.05 (15)	O6—C14—C13	111.21 (16)
C3—C4—C5	116.51 (14)	C16A—C15A—O6	100.0 (12)
C3—C4—H4	121.7	C16A—C15A—H15A	111.8
C5—C4—H4	121.7	O6—C15A—H15A	111.8
C7—C5—C4	134.93 (14)	C16A—C15A—H15B	111.8
C7—C5—C6	104.42 (12)	O6—C15A—H15B	111.8
C4—C5—C6	120.65 (13)	H15A—C15A—H15B	109.5
N2—C6—C1	127.71 (14)	C15A—C16A—H16A	109.5
N2—C6—C5	111.56 (13)	C15A—C16A—H16B	109.5
C1—C6—C5	120.72 (14)	H16A—C16A—H16B	109.5
N3—C7—C5	106.23 (12)	C15A—C16A—H16C	109.5
N3—C7—H7	126.9	H16A—C16A—H16C	109.5
C5—C7—H7	126.9	H16B—C16A—H16C	109.5
N3—C8—C9	109.71 (12)	O6—C15B—C16B	107.7 (4)
N3—C8—C13	108.61 (11)	O6—C15B—H15C	110.2
C9—C8—C13	112.54 (12)	C16B—C15B—H15C	110.2
N3—C8—C17	105.09 (11)	O6—C15B—H15D	110.2
C9—C8—C17	112.63 (12)	C16B—C15B—H15D	110.2
C13—C8—C17	107.94 (13)	H15C—C15B—H15D	108.5
C10—C9—C8	113.73 (13)	C15B—C16B—H16D	109.5
C10—C9—H9A	108.8	C15B—C16B—H16E	109.5
C8—C9—H9A	108.8	H16D—C16B—H16E	109.5
C10—C9—H9B	108.8	C15B—C16B—H16F	109.5
C8—C9—H9B	108.8	H16D—C16B—H16F	109.5
H9A—C9—H9B	107.7	H16E—C16B—H16F	109.5
O3—C10—O4	123.57 (17)	O7—C17—O8	125.39 (16)
O3—C10—C9	125.79 (15)	O7—C17—C8	121.80 (14)
O4—C10—C9	110.64 (14)	O8—C17—C8	112.81 (14)
O4—C11A—C12A	99.7 (9)	C19—C18—O8	111.62 (18)
O4—C11A—H11A	111.8	C19—C18—H18A	109.3
C12A—C11A—H11A	111.8	O8—C18—H18A	109.3
O4—C11A—H11B	111.8	C19—C18—H18B	109.3
C12A—C11A—H11B	111.8	O8—C18—H18B	109.3
H11A—C11A—H11B	109.5	H18A—C18—H18B	108.0
C11A—C12A—H12A	109.5	C18—C19—H19A	109.5
C11A—C12A—H12B	109.5	C18—C19—H19B	109.5
H12A—C12A—H12B	109.5	H19A—C19—H19B	109.5
C11A—C12A—H12C	109.5	C18—C19—H19C	109.5
H12A—C12A—H12C	109.5	H19A—C19—H19C	109.5
H12B—C12A—H12C	109.5	H19B—C19—H19C	109.5
C12B—C11B—O4	107.5 (7)	O1—N1—O2	122.96 (16)
C12B—C11B—H11C	110.2	O1—N1—C3	119.26 (15)
O4—C11B—H11C	110.2	O2—N1—C3	117.76 (16)
C12B—C11B—H11D	110.2	C6—N2—N3	103.56 (12)
O4—C11B—H11D	110.2	C7—N3—N2	114.22 (12)
H11C—C11B—H11D	108.5	C7—N3—C8	129.31 (12)
C11B—C12B—H12D	109.5	N2—N3—C8	116.45 (11)
C11B—C12B—H12E	109.5	C10—O4—C11A	120.8 (5)
H12D—C12B—H12E	109.5	C10—O4—C11B	111.8 (3)
C11B—C12B—H12F	109.5	C14—O6—C15B	122.4 (4)
H12D—C12B—H12F	109.5	C14—O6—C15A	102.2 (4)
H12E—C12B—H12F	109.5	C17—O8—C18	116.58 (15)

### Hydrogen-bond geometry (Å, º)

**Table:**

*D*—H···*A*	*D*—H	H···*A*	*D*···*A*	*D*—H···*A*
C1—H1···N2^i^	0.93	2.53	3.443 (2)	168
C4—H4···O7^ii^	0.93	2.53	3.205 (2)	129

Symmetry codes: (i) −*x*+1, −*y*+1, −*z*+1; (ii) *x*, −*y*+1/2, *z*−1/2.
