# Supplementary figures and images for: Crystal structure of triethyl 2-(5-nitro-2H-indazol-2-yl)propane-1,2,3-tri­carboxyl­ate
Source: Acta Crystallogr E Crystallogr Commun. 2015 Sep 26;71(Pt 10):o780–1. doi: 10.1107/S2056989015017235 (PMC4647391; doi:10.1107/S2056989015017235)

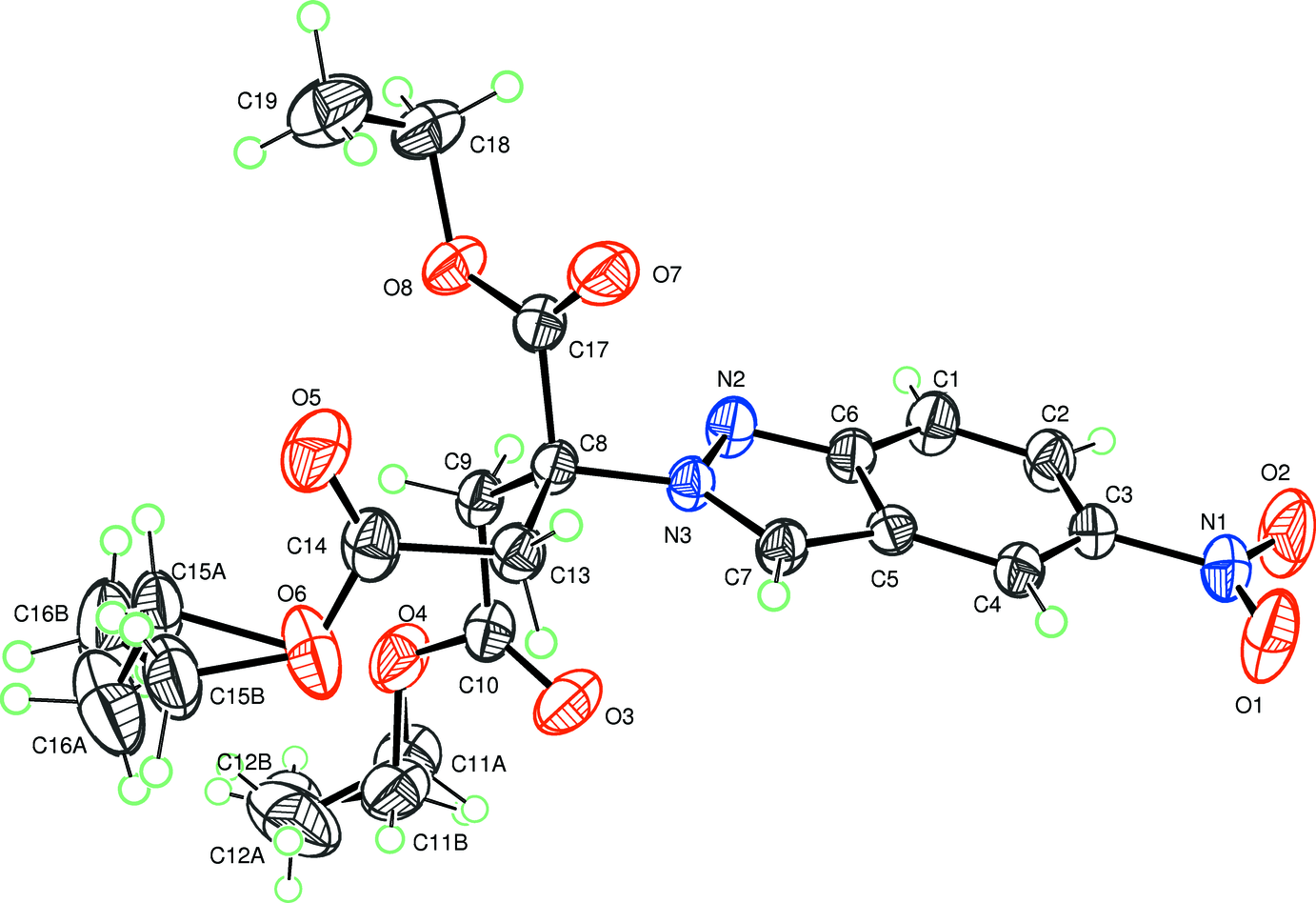

Supplement: Supplementary file 4 [file e-71-0o780-fig1.tif]

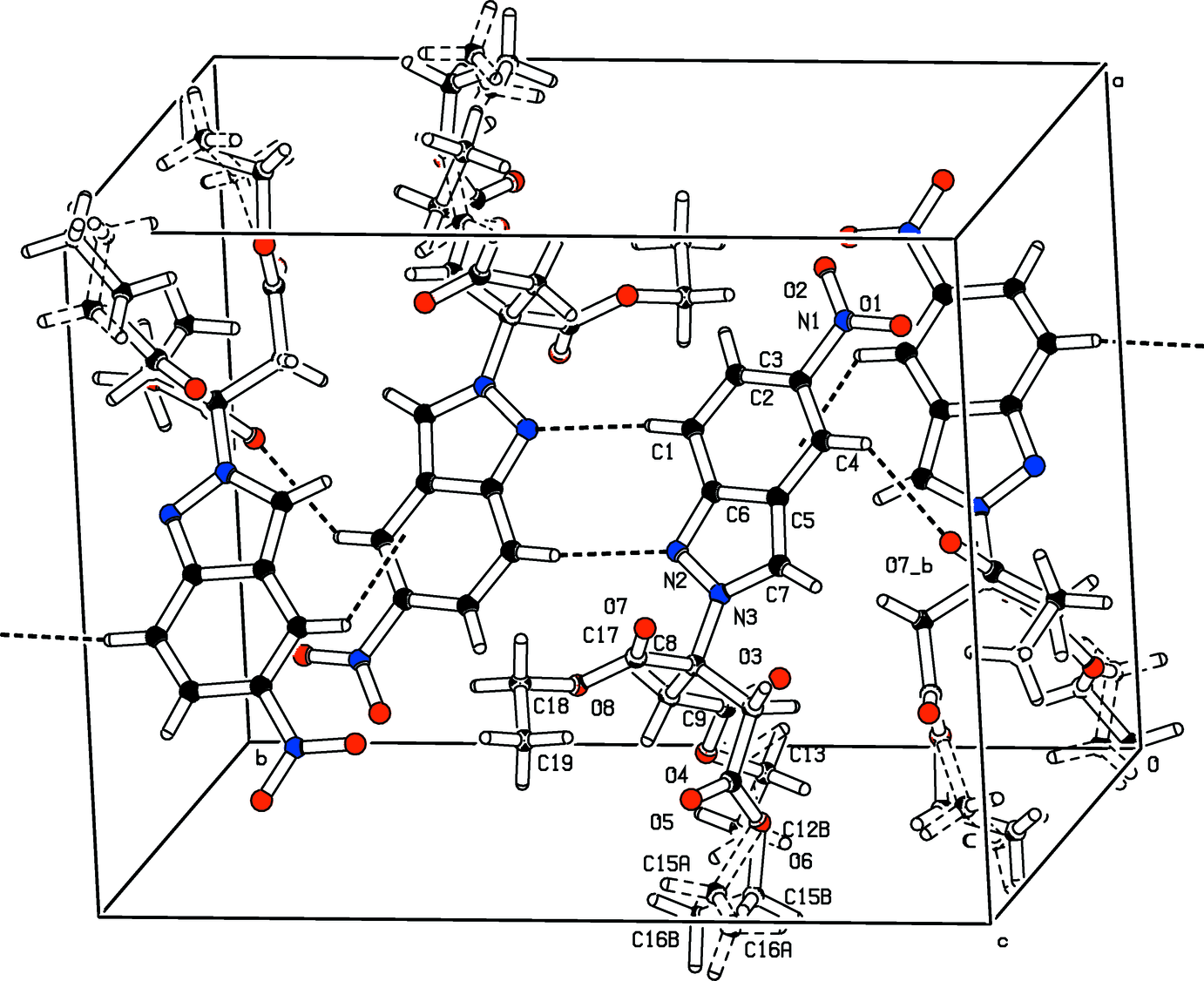

Supplement: Supplementary file 5 [file e-71-0o780-fig2.tif]
